# Effect of curcumin on rheumatoid arthritis: a systematic review and meta-analysis

**DOI:** 10.3389/fimmu.2023.1121655

**Published:** 2023-05-31

**Authors:** Haiyang Kou, Lei Huang, Meng Jin, Qi He, Rui Zhang, Jianbing Ma

**Affiliations:** ^1^ Department of Joint Surgery, Translational Medicine Center, Honghui Hospital, Xi’an Jiaotong University, Xi’an, Shaanxi, China; ^2^ Department of Medical Technology, Guiyang Healthcare Vocational University, Guiyang, Guizhou, China

**Keywords:** curcumin, rheumatoid arthritis, arthritis, inflammatory, antioxidants

## Abstract

**Objective:**

The aim of this study is to evaluate the effectiveness and safety of curcumin in rheumatoid arthritis patients.

**Methods:**

A computerized search from PubMed, Embase, Cochrane Library, and Web of Science databases was performed until 3 March 2023. Literature screening, basic data extraction and risk of bias evaluation were independently performed by two researchers each. The quality evaluation of the literature was performed according to the Cochrane Handbook for Risk of Bias Assessment tool for treatment evaluation.

**Results:**

The current study includes six publications covering 539 rheumatoid arthritis patients. The activity of rheumatoid arthritis was assessed using erythrocyte sedimentation rate (ESR), C-reactive protein (CRP), protein, disease activity score (DAS), rheumatoid factor (RF), Visual Analogue Scale (VAS) pain, tender joint count (TJC) and swollen joint count (SJC). ESR (MD = -29.47, 95% CI [-54.05, -4.88], Z=2.35, P = 0.02), DAS28 (MD = -1.20, 95% CI [-1.85, -0.55], Z=3.62, P = 0.0003), SJC (MD = -5.33, 95% CI [-9.90, -0.76], Z = 2.29, P = 0.02) and TJC (MD = -6.33, 95% CI [-10.86, -1.81], Z = 2.74, P = 0.006) showed significantly change in experimental patients compared with controls.

**Conclusion:**

Curcumin is beneficial for rheumatoid arthritis treatment. Inflammation levels and clinical symptoms in patients with rheumatoid arthritis can be improved by curcumin supplementation. Large sample randomized controlled trials on the effects of curcumin on patients with rheumatoid arthritis are needed in the future.

**Systematic review registration:**

https://www.crd.york.ac.uk/PROSPERO/, identifier (CRD42022361992).

## 1 Introduction

Rheumatoid arthritis (RA) is a common autoimmune arthritis disease, characterized by a chronic recurrent symmetrical polyarthritis (1). In most cases, rheumatoid arthritis has an insidious onset with one or more swelling joints at the beginning of the disease (2). Proximal interphalangeal, metacarpophalangeal, wrist and foot joints are more common to suffer from swelling (3). Rheumatoid arthritis patients also have symptomatic joint congestion and edema, tenderness, stiffness, joint pain. Most joint swelling and redness occur in the morning and evening in acute attacks, fluid exudation occurs in chronic phase, and joint muscle atrophy and joint deformity are likely accompanied in the late stage of the disease (4). In addition, rheumatoid arthritis can lead to some complications such as fever, insomnia, anxiety, depression, pulmonary fibrosis, anemia, leukopenia, ocular sclerosis, leg ulcers (5). Chronic inflammation in rheumatoid arthritis leads to damage to cartilage and bone, resulting in cartilage erosion, bone degeneration, and joint deformity and loss of range of motion (6). One study in Danish adults reported that the average prevalence and incidence of rheumatoid arthritis was approximately twice as high in women as in men (7). Rheumatoid arthritis not only bring physical harm, but also affect mental health (8). The disease leads to unbearable repeated pain and endless medication to patients and is also known as the “invisible killer”. Nevertheless, rheumatoid arthritis can achieve good control of symptoms and avoid joint deformity by early diagnosis and treatment (9).

Rheumatoid arthritis is very ‘sneaky’, often undetectable and easily confused with other diseases in its early stage, and can get progressively worse over time (10). The clinical treatment for rheumatoid arthritis includes pain medication, physical therapy and, in some cases, essential surgery. The existing disease-modifying antirheumatic drugs (DMARDs) can be broadly divided into three categories: conventionally synthesized DMARDs (e.g., methotrexate), targeted synthetic DMARDs (e.g., pan-JAK and JAK1/2 inhibitors), and biological DMARDs (e.g., (TNF) -α inhibitors, IL-6R inhibitors) (11). DMARDs for rheumatoid arthritis treatment have the potential to greatly improve disease symptoms and prevent disease progression. Meanwhile, DMARDs produce gastrointestinal toxicity and side effects of cardiovascular disease and result in high economic costs (12). Different formulations of traditional Chinese medicine (THM) have been shown to have anti-rheumatoid arthritis effects and improve some symptoms. The traditional Chinese medicine prescriptions for rheumatoid arthritis have the potential to improve the symptoms and delay the progression of this chronic disease (13).

Turmeric is a perennial herb belonging to the genus Curcuma in the family Zingiberaceae. Curcumin is the most important chemical component of turmeric, which can exert antioxidant, anti-inflammatory, anti-angiogenic and anti-tumor pharmacological effects without significant adverse effects (14). Previous studies have shown that curcumin and curcuminoids in turmeric could provide good protection against many chronic diseases in the body by inhibiting inflammatory responses, lowering blood lipids, and improving blood sugar (15). Curcumin in turmeric can effectively inhibit inflammatory reactions and reduce symptoms such as pain and swelling. In recent years, it was found that curcumin could alleviate some symptoms in some autoimmune diseases such as rheumatoid arthritis and inflammatory bowel disease (16). The researchers have conducted numerous studies to evaluate the pharmacological effects and clinical applications of turmeric and curcumin on rheumatoid arthritis treatment (17).

In this systematic review and meta-analysis, we investigated the effectiveness and safety of curcumin on the treatment of rheumatoid arthritis based on 10 randomized controlled trials (RCTs). We summarized and analyzed both laboratory test factors and clinical test indicators to evaluate the anti-inflammation and immunomodulatory function of supplementary curcumin on rheumatoid arthritis treatment.

## 2 Methods

### 2.1 Search strategy

This systematic review was conducted according to the Preferred Reporting Items guidelines for systematic reviews and meta-analyses. A computerized search from PubMed, Embase, Cochrane Library, and Web of Science databases was performed until 3 March 2023. The quality evaluation of the literature was performed according to the Cochrane Handbook for Risk of Bias Assessment tool for treatment evaluation. This systematic review has been prospectively registered on the ROSPERO website under the ID number CRD42022361992.The computerized search is constructed by combining subject headings and free words. According to different databases, manual searches combined subject headings and keywords with relevant references. The search key words are: ((“Arthritis, Rheumatoid”[Mesh]) OR (Rheumatoid Arthritis[Title/Abstract])) AND ((“Curcumin”[Mesh]) OR (1,6-Heptadiene-3,5-dione, 1,7-bis(4-hydroxy-3-methoxyphenyl)-, (E,E)-[Title/Abstract]) OR (Turmeric Yellow[Title/Abstract]) OR (Yellow, Turmeric[Title/Abstract]) OR (Curcumin Phytosome[Title/Abstract]) OR (Phytosome, Curcumin[Title/Abstract]) OR (Diferuloylmethane[Title/Abstract]) OR (Mervia[Title/Abstract])) using Boolean operators.

### 2.2 Study selection

The imported literature was checked and selected by Endnote literature management software. Literature screening, basic data extraction and risk of bias evaluation were independently performed by two researchers following the established inclusion and exclusion criteria. In case of disagreement, they cross-checked the information through mutual discussion or based on third-party rulings.

### 2.3 Inclusion criteria

(1) Study design. Randomized controlled trial of clinical trial study. (2) Study subjects. Patients with rheumatoid arthritis who met the diagnostic criteria for index impairment regardless of age, sex, or race. (3) Interventions. Curcumin supplements were administered to the treatment group; non-curcumin supplements, such as standard clinical treatment, or placebo, were administered to the control group. (4) Results-based measures. The primary outcome measure was the symptom resolution or functional score improvement after medication. The secondary outcome measures were some adverse effects.

### 2.4 Exclusion criteria

(1) RCT patients with rheumatoid arthritis combined with knee osteoarthritis or other joint diseases. (2) The types of experimental studies are pharmacological tests, animal experiments or cohort studies. (3) Patients with severe heart, brain, kidney disease and malignant tumors; (4) Case reports, conference papers and related reviews. (5) Publications with repeated indicators. (6) Data error, unable to obtain full text of literature. (7) Literature with incomplete outcome measures or literature without outcome measures.

### 2.5 Quality assessment

The Newcastle Ottawa scale (NOS) was used to evaluate the quality of the literature with 3 items and 9 sub-items (18). The evaluation of the scores was classed into four types: 4 points for cases and controls involving the sufficient data of the case, the representativeness of the case, the selection of the control and the definition of the control), 3 points for the exposure including the determination of the exposure, whether the same determination method was used for the exposure of the case and the control, and the non-response rate, and 2 points for the comparability of the case and the control based on design or analysis. The full score was 9 points, and ≥ 5 points means high-quality literature.

### 2.6 Bias assessment

The methodological quality of the included articles were evaluated according to the “Bias Risk Assessment” tool in Cochrane Reviewer’s Handbook 5.1 part. Finally, three quality results of the literature were judged, including “low risk of bias”, “high risk of bias” and “uncertain risk of bias”. The methodological quality of the included articles was independently assessed and cross-checked by two investigators to assess the risk of bias. They consult a third party when questions or opinions were inconsistent.

### 2.7 Data extraction

The data were independently screened, extracted and cross-checked the literature by two researchers, and those with different opinions were handed over to a third party for consultation. The extracted data include: (1) basic information of patients (author, year, etc.); (2) baseline characteristics and interventions of study subjects; (3) key content of risk of bias assessment; (4) outcome measures of interest and data results. If necessary, they contacted the original author for relevant missing data.

### 2.8 Statistical analysis

RevMan 5.4 and Stata 17 software were used to analyze the data. The q-test was used to assess study heterogeneity. The fixed-effect model was selected to analyze study data in case of *P*>0.1 and I^2^ ≤ 50%, indicating homogeneity among the studies. Conversely, *P*<0.1 and I^2^>50% indicated that the heterogeneity of the studies was large and the random-effects model was selected to analyze the studies. The standardized mean difference (SMD) was used to analyze continuous variables, and odds ratio (OR) was used to analyze dichotomous variables. Each effect measure was expressed with 95% confidence interval (CI). Stata 17.0 was used to plot publication bias, and Review Manager 5.4.1 software was used to evaluate the quality of the literature.

## 3 Results

### 3.1 Search results

**Figure 1:**
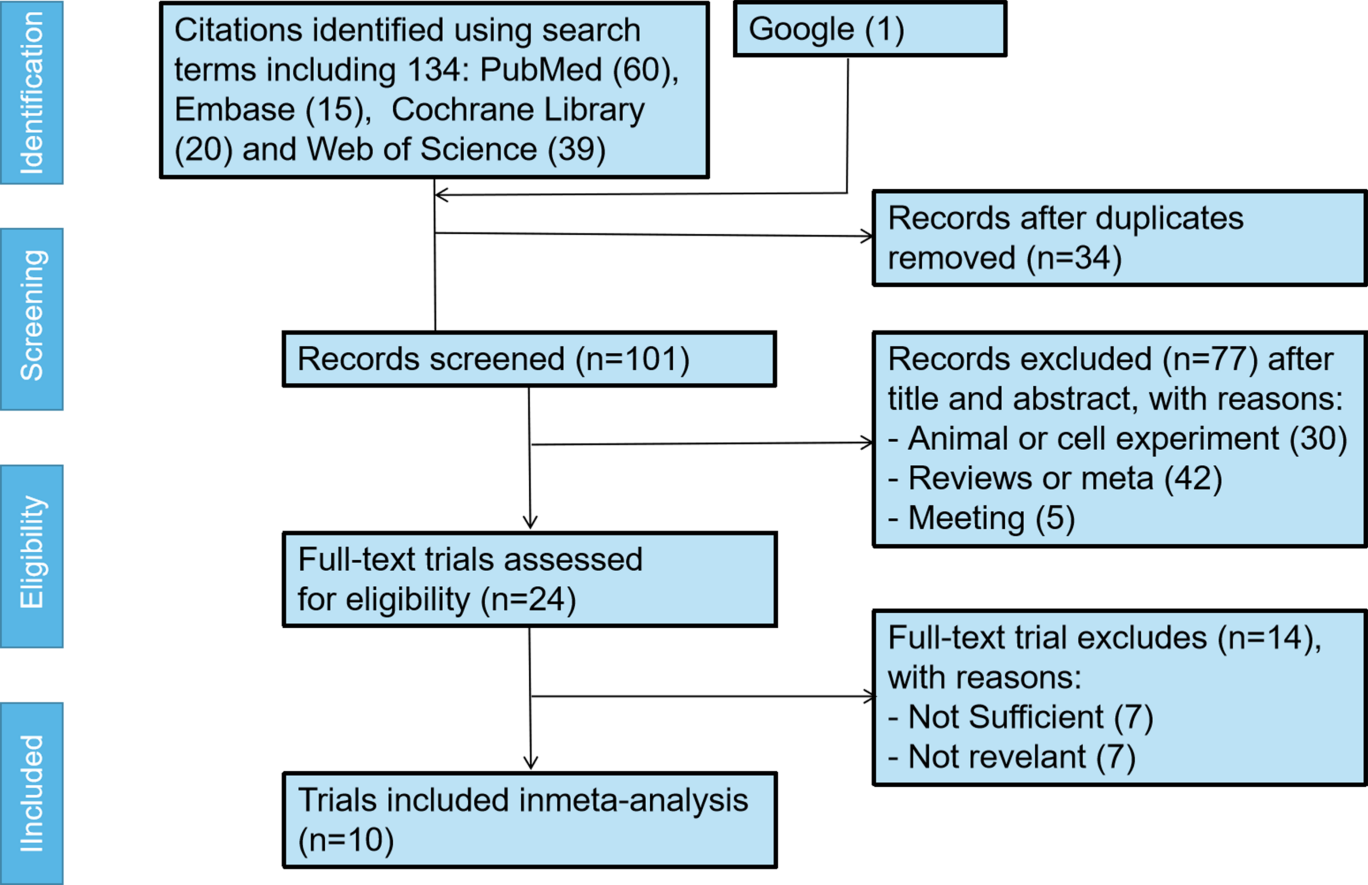
Flow chart of the study’s screening process. RCT, randomized controlled trial.

A total of 135 articles were retrieved from four databases, with 20, 60, 15, 39 and 1 articles in the Cochrane Library, PubMed, Embase, Web of Science and google respectively. After checking the titles and abstracts, articles not related to the topic were excluded and the remaining 101 articles were fully checked for further analysis. Seventy-seven interventions that did not meet the inclusion criteria and 14 articles with unclear results were excluded. Finally, 10 articles were included. The specific screening process was shown in **Figure 1**.

### 3.2 Study characteristics

The screened ten clinical trials included 539 patients, mainly from Middle East and South Asia, small section from China (19–28). Curcumin was administered to the treatment group, while other drugs or placebo were administered to the control group. The basic characteristics of the included studies are presented in **Table 1**.

**Table 1:** Baseline characteristics of curcumin study participants.

Reference	No.	Age	Females	Areas	Treatment Strategies	Outcomes	Quality score
A.Amalraj2017(21)	36	38.2 (8.5)	15 (41.7%)	India	Twelve patients in each group received placebo, 250 or 500 mg of the curcumin product twice daily for 90 days.	①②③④ ⑤⑥⑦⑨	8
B.Chandran2012(22)	45	47.88 (12.03)	38 (84.4%)	India	Forty-five patients diagnosed with RA were randomized into three groups with patients receiving curcumin (500 mg) and diclofenac sodium (50 mg) alone or their combination.	①②③④ ⑤⑥	7
F.P.Zarandi2022(23)	44	50.52 (9.7)	44 (100%)	Iran	The patients were treated with curcumin (500 mg once a day) or placebo for 8 weeks.	④⑤	6
M.Javadi2019(24)	49	55.02 (2.9)	44 (89.8%)	Iran	Curcumin nanomicelle (40 mg) and placebo capsules were administrated to the RA patients 3 times a day for 12 weeks.	①④⑥⑦	6
S.D.Deodhar1980(25)	18	36.3	16 (88.9%)	India	18 patients with 'definite' rbeumatoid arthritis to compare the antirbeumatic activity of curcumin (diferuloyl methane) 1200,mg/day with phenylbutazone 300mg/day.	④⑥	6
A.A.Hemmati2016(26)	60	NR	NR	Iran	One group received compound herbal drug named Curcumex consist of and control group received placebo as a same dose.	①④⑤⑥ ⑦	7
M.K.Khan2022(27)	90	45-60 years	NR	India	Ninety patients were randomised into two groups. Curcumin dosed at 180 mg/day was given orally to both groups. The treatment regimen was distributed as 3 sessions/week.	④⑤⑨	6
D.Anusha2019(28)	45	42.9 (4.8)	45 (100%)	Iran	A triple−blinded controlled trial was conducted among 45 female RA patients with CP randomized into three treatment groups as follows.	④⑤⑨	6
J.Lin(29)	128	46.7 (7.9)	65 (50.8%)	China	RA patients were randomly divided into control and treatment groups, with the control group receiving oral methotrexate and the treatment group receiving methotrexate combined with oral curcumin.	①	5
J.Jacob(30)	24	45-60 years	12 (50%)	India	Patients were randomized in 1:1:1 ratio to receive 250 mg, 500 mg curcumin or placebo as one capsule a day, over a period of three months.	①②③④ ⑤	7

BMI, body mass index; NR, not reported. Outcomes: ① DAS28, Disease Activity Score; ② ACR, American College of Rheumatology; ③ VAS, Visual analogue scale; ④ ESR, erythrocyte sedimentation rate; ⑤ CRP, C-reactive protein; ⑥ Swollen joint count; ⑦ Tender joint count; ⑧ Morning stiffness; ⑨ RF, Rheumatoid Factor.

### 3.3 Risk of bias assessment

**Figure 2:**
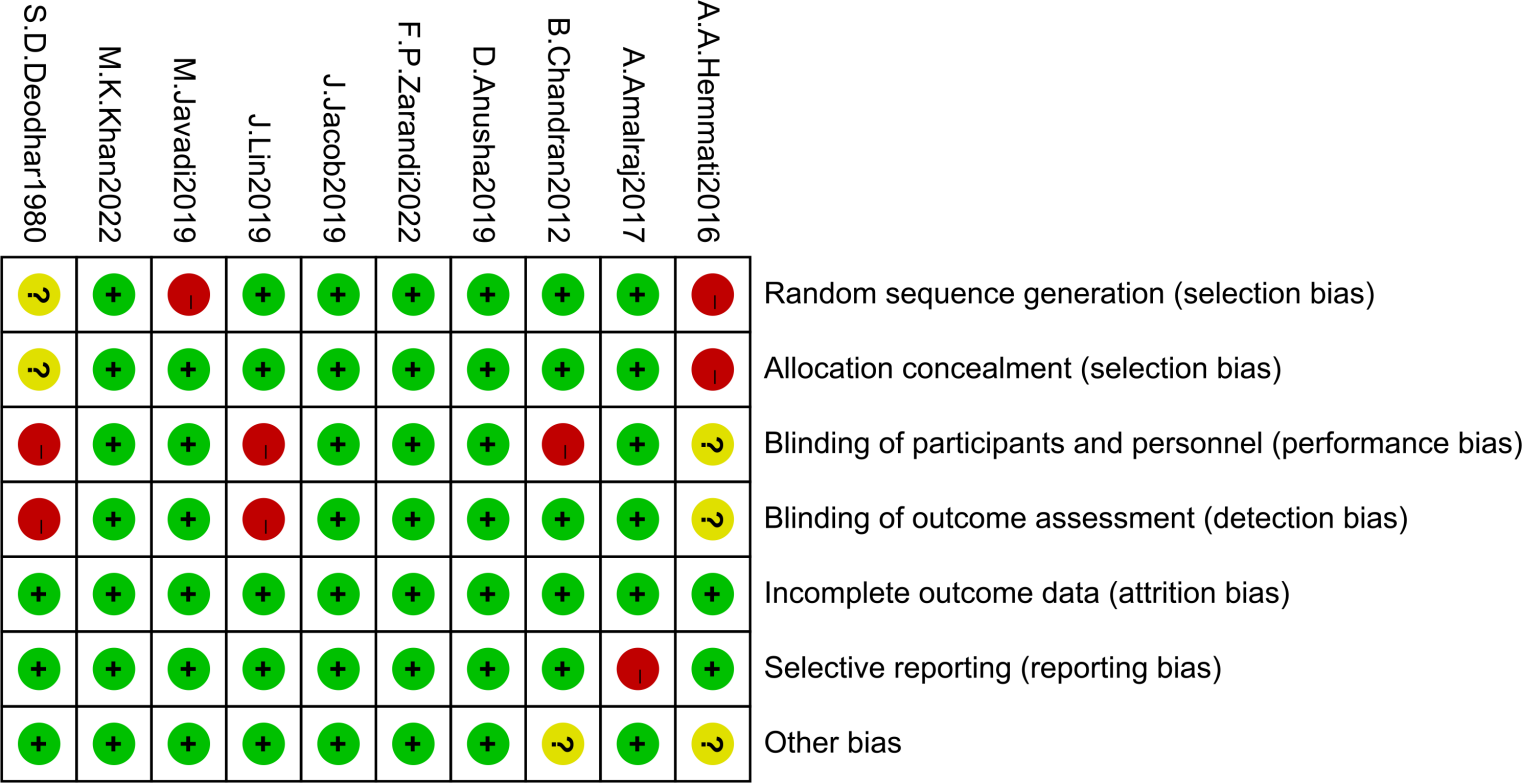
Risk of bias in the selected studies.

The risk of bias assessment for the included articles was shown in **Figure 2**. This systematic assessment and meta-analysis risk of bias was assessed as moderate, with 2 studies with random sequence generation as highly biased and 1 unknown, 1 study with allocation concealment as highly biased and 1 unknown, 3 studies with blinding of participants and personnel as highly biased and 1 unknown, 2 studies blinding of outcome assessment as highly biased and 1 unknown, 1 study with selective reporting as highly biased, no study with incomplete outcome data, and 2 other studies with bias unknown.

### 3.4 Efficacy

#### 3.4.1 Changes from controls in laboratory measurements

**Figure 3:**
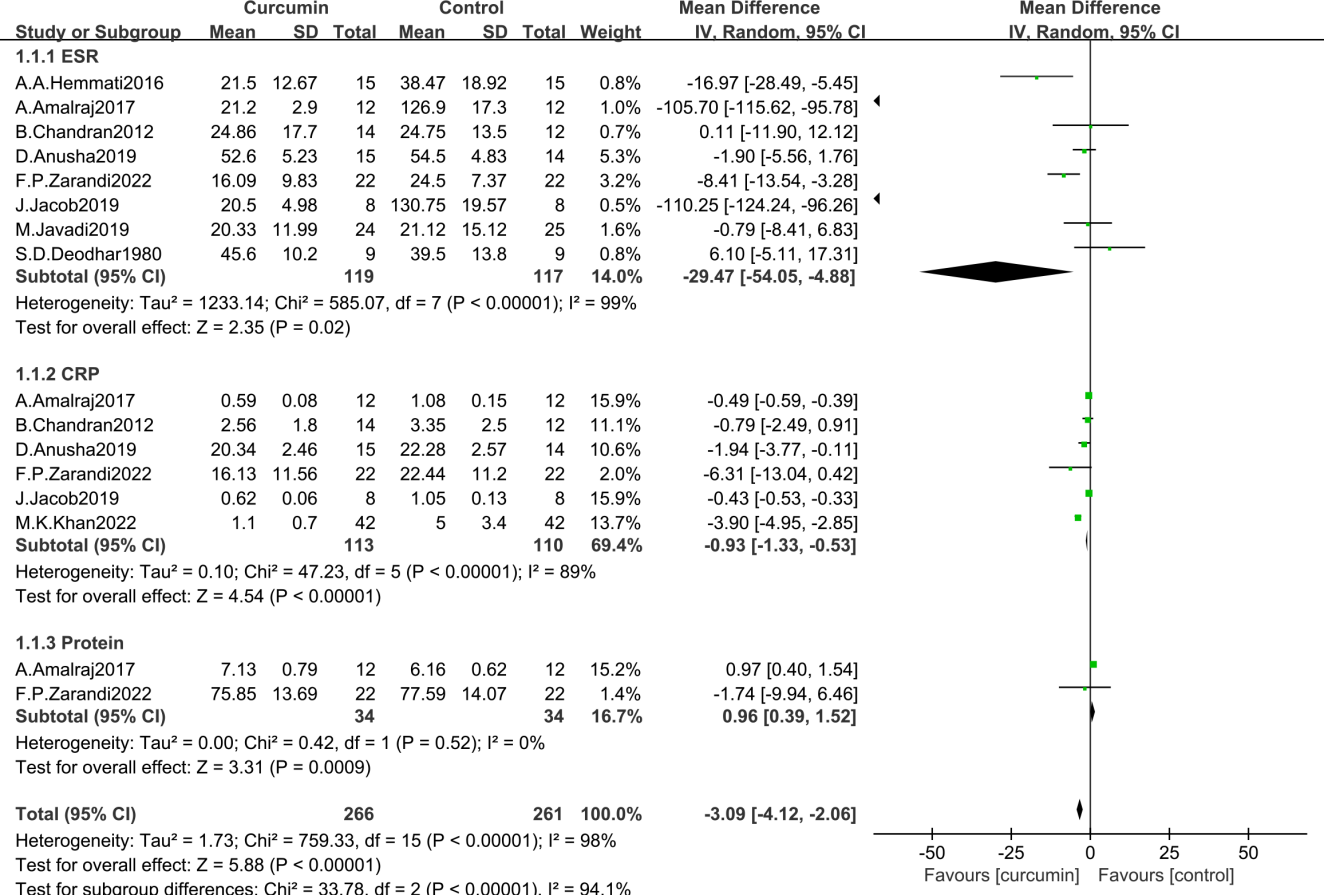
Effect of curcumin and placebo in the comparison of laboratory measurements in patients with RA.

Nine trials were conducted using a meta-analysis to determine the mean change in the usage of curcumin in rheumatoid arthritis patients (19–26, 28) and the results were shown in **Figure 3**. The results of heterogeneity analysis showed that statistical heterogeneity of the laboratory test factors was existed in the studies and therefore the random effects model was used. In comparison to the control group, the effect of ESR (MD = -29.47, 95% CI [-54.05, -4.88], Z = 2.35, P = 0.02; **Figure 3**) and CRP (MD = -0.93, 95% CI [-1.33, -0.53], Z = 4.54, P < 0.00001; **Figure 3**) was significantly different in patients in the experimental group. Rheumatoid arthritis patients who take curcumin have improved greatly in ESR and CRP. The effect of curcumin on total protein (MD = 0.96, 95% CI [0.39, 1.52], Z = 3.31, P = 0.0009; **Figure 3**) in cases and controls was only reported by two studies (17, 19). The overall effect of the laboratory test factors was improved for rheumatoid arthritis patients.

#### 3.4.2 Change from control in clinical measurements

**Figure 4:**
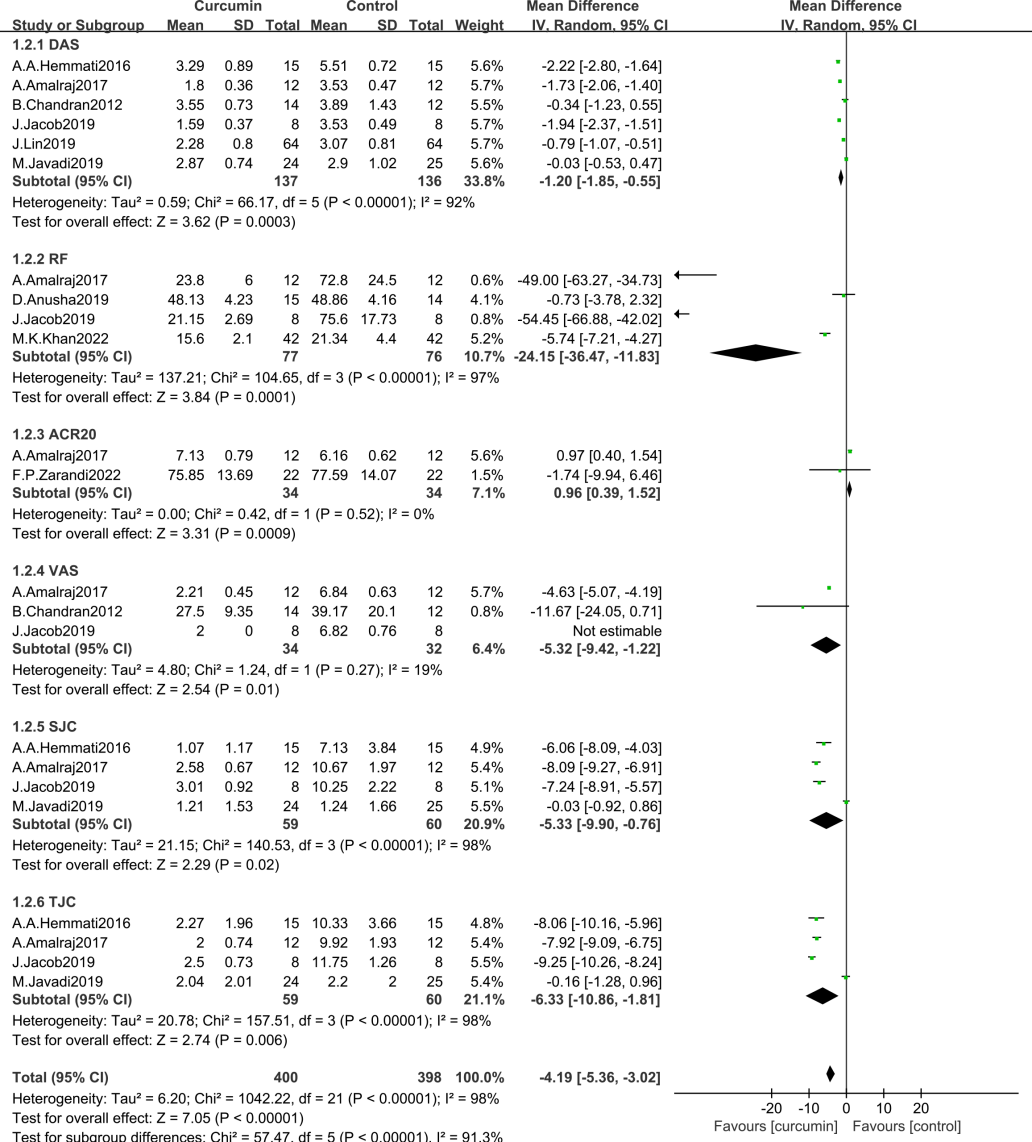
Effectiveness of curcumin and placebo in the comparison of clinical measures in patients with RA.

A meta-analysis evaluating the mean change in the usage of curcumin in patients with rheumatoid arthritis included eight trials (experimental group (n = 137) and the control group (n = 136)) (19, 20, 22, 24–28). The heterogeneity analysis showed that there was statistical heterogeneity in the clinical test indicators (**Figure 4**). The results of effect test showed that the favor indicators of curcumin for the rheumatoid arthritis include: DAS28 (MD = -1.20, 95% CI [-1.85, -0.55], Z = 3.62, P = 0.0003; **Figure 4**), RF (MD = -24.15, 95% CI [-36.47, -11.83], Z = 3.84, P = 0.0001; **Figure 4**), VAS (MD = -5.32, 95% CI [-9.42, -1.22], Z = 2.54, P = 0.01; **Figure 4**), SJC (MD = -5.33, 95% CI [-9.90, -0.76], Z = 2.29, P = 0.02; **Figure 4**) and TJC (MD = -6.33, 95% CI [-10.86, -1.81], Z = 2.74, P = 0.006; **Figure 4**). Rheumatoid arthritis patients in the experimental group added with curcumin did not gain more benefit ACR20 (MD = 0.96, 95% CI [0.39, 1.52], Z=3.31, P = 0.0009; **Figure 4**) than those in the control group. The overall effect of the clinical test indicators was improved for rheumatoid arthritis patients.

#### 3.4.3 Meta-analysis of subgroups of changes in clinical and laboratory indicators

Stratification of eligible studies was conducted to three groups including Indians (5 studies), Iranians (4 studies) and Chinese (1study). The results of heterogeneity analysis of combined CRP in Iranians and combined SJC in Indian was homogeneity (**Supplementary Figures 1–3**). The results of pooled ORs, heterogeneity and publication bias analysis in these subgroups were shown in **Table 2**. There was less heterogeneity in the subgroups compared with overall results. In addition, meta-regression analysis was employed to investigate the effect of total score (NOS) on heterogeneity, and the results indicate that the methodological quality of the study did not affect the results (**Table 3**).

**Table 2:** Heterogeneity and publication bias of curcumin and placebo in patients with RA.

Subgroup	Model	Sample size	Test of association		Test of heterogeneity		Test of publication bias (Begg's test)		Test of publication bias (Egger's test)	
	Metrics	Case/control	OR	95%CI (p-value)	I^2^ (%)	p	z	p	t	P
Overall	ESR	119/117	-29.47	-54.05–-4.88 (p< 0.001)	98.8	< 0.001	1.11	0.266	-1.48	0.188
	CRP	113/110	-0.93	-1.33–-0.53 (p< 0.001)	89.4	< 0.001	1.13	0.260	-2.04	0.111
	DAS28	137/136	-1.20	-1.85–-0.55 (p< 0.001)	92.4	< 0.001	-0.19	0.851	0	0.999
	RF	77/76	-24.15	-36.47–-11.83 (p< 0.001)	97.1	< 0.001	0.34	0.734	-1.79	0.215
	ACR20	20/20	53.41	48.70–58.11 (p< 0.001)	0	0.87	1.00	0.317	0	0
	VAS pain	26/24	-5.32	-9.42–-1.22 (p< 0.001)	19.4	0.265	-1.00	0.317	0	0
	TJC	51/52	-5.35	-11.01–0.31 (p< 0.001)	98	< 0.001	-0.52	0.602	-0.47	0.722
	SJC	51/52	-4.70	-10.43–1.029 (p< 0.001)	98.4	< 0.001	-0.52	0.602	-0.74	0.594
	protein	34/34	0.96	0.39–1.52 (p< 0.001)	0	0.518	-1.00	0.317	0	0
Subgroup (Ethnicity or Country)										
India	ESR	43/41	-52.41	-115.64–10.82 (p< 0.001)	99.2	< 0.001	-0.34	1	0.10	0.928
	CRP	76/74	-0.85	-1.24–-0.45 (p< 0.001)	92.8	< 0.001	-0.34	1	-1.49	0.275
	DAS28	34/32	-1.48	-2.12–-0.83 (p< 0.001)	80.2	0.006	0.52	0.602	1.71	0.337
	RF	44/44	-35.97	-73.14–1.21 (p< 0.001)	97.8	< 0.001	-0.52	0.602	-7.23	0.087
	VAS pain	26/24	-5.32	-9.42–-1.22 (p< 0.001)	19.4	0.265	-1.00	0.317	0	0
	TJC	12/12	-7.92	-9.09–-6.75 (p< 0.001)	0	< 0.001	0	0	0	0
	SJC	12/12	-8.09	-9.27–-6.91 (p< 0.001)	0	< 0.001	0	0	0	0
	protein	12/12	0.97	0.40–1.54 (p< 0.001)	0	0	0	0	0	0
Iran	ESR	76/76	-5.76	-11.31–-0.21 (p< 0.001)	68.6	0.023	1.02	0.308	-1.08	0.394
	CRP	37/36	-2.88	-6.39–0.64 (p< 0.001)	33.8	0.219	-1.00	0.317	0	0
	DAS28	39/40	-1.12	-3.27–1.03 (p< 0.001)	96.8	< 0.001	-1.00	0.317	0	0
	RF	15/14	-0.73	-3.78–2.32 (p< 0.001)	0	0	0	0	0	0
	TJC	39/40	-4.06	-11.80–3.68 (p< 0.001)	97.6	< 0.001	-1.00	0.317	0	0
	SJC	39/40	-2.97	-8.88–2.93 (p< 0.001)	96.5	< 0.001	1	0.317	0	0
	protein	22/22	-1.74	-9.94–6.46 (p< 0.001)	0	0	0	0	0	0
China	DAS28	64/64	-0.79	-1.07–-0.51 (p< 0.001)	0	0	0	0	0	0

**Table 3:** Meta-regression of curcumin and placebo in patients with RA.

Heterogeneity factors (NOS score)	Coefficient	Std. err.	z	p	[95% conf. interval]	
					LL	UL
ESR	-29.59	17.18	-1.72	0.09	-63.27	4.09
CRP	-0.48	0.04	-13.64	0.00	-0.55	-0.41
protein	0.96	0.29	3.31	0.01	0.39	1.52
DAS28	-1.19	0.37	-3.26	0.00	-1.91	-0.48
RF	-26.80	14.08	-1.90	0.06	-54.40	0.79
ACR20	53.41	2.40	22.26	0.00	48.70	58.11
VAS pain	-4.64	0.22	-20.77	0.00	-5.08	-4.20
TJC	-5.34	2.63	-2.03	0.04	-10.49	-0.20
SJC	-4.70	2.45	-1.91	0.06	-9.50	0.11

### 3.5 Sensitivity analysis and meta-analysis of publication bias

**Figure 5:**
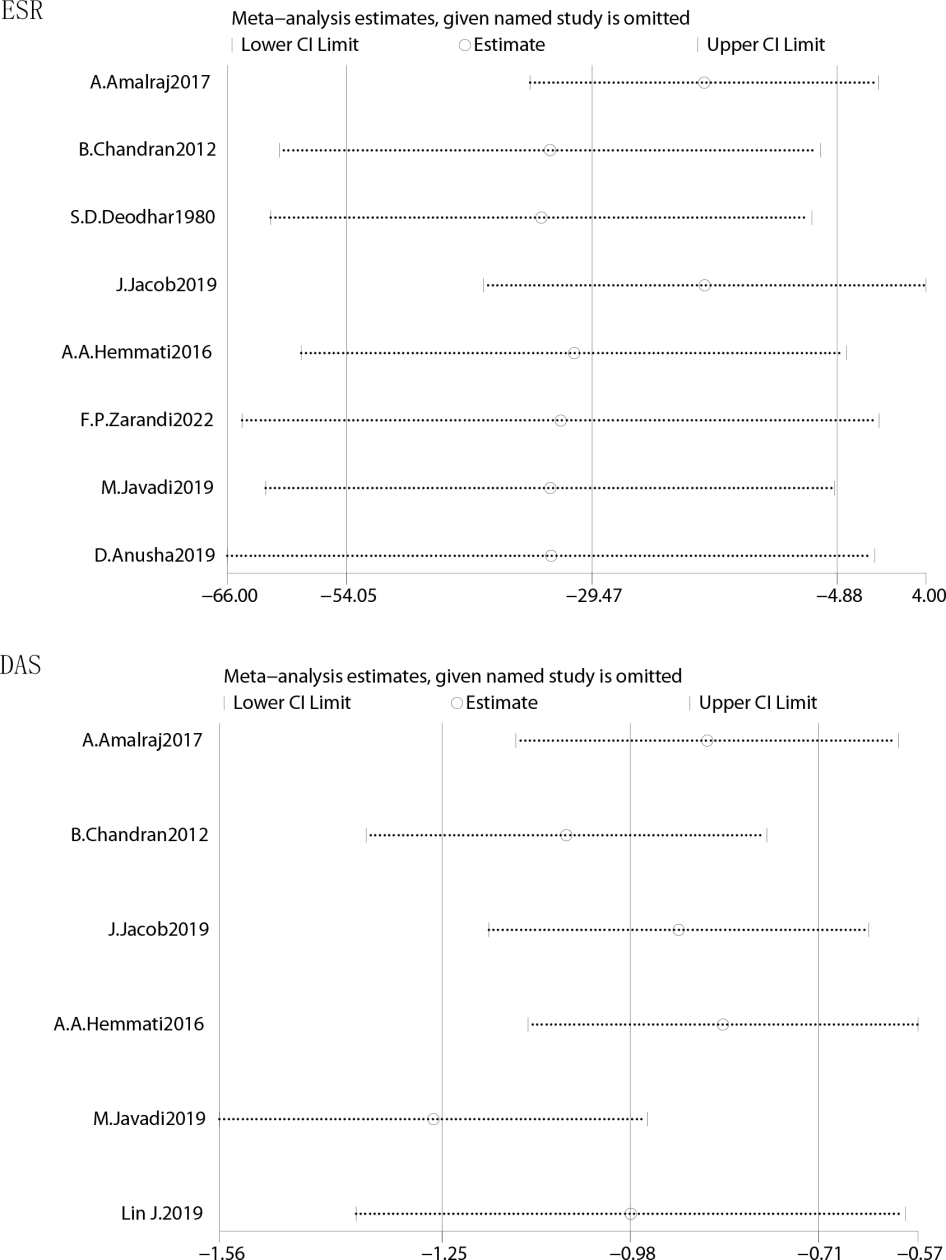
Sensitivity of curcumin and placebo in patients with RA.

The effect data were conducted in sensitivity and publication bias analyses. The results of overall sensitivity analyses demonstrated that the results of ESR, CRP and DAS were robust and omitting one of studies in these three test factors would not significantly affect the overall measured outcome (**Figure 5**). The results of funnel plot showed that there was no significant evidence of asymmetry in the shape of the funnel plot, indicating no publication bias (**Supplementary Figure 4**). The results from Begg’s test and Egger’s tests in **Table 2** indicated that there was no publish bias.

### 3.6 Safety

**Figure 6:**
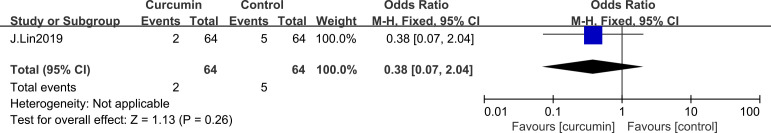
Safety of curcumin and placebo in patients with RA.

In these ten studies, only one trial reported slight adverse events such as signs of rash, diarrhea, and vomiting. The fixed model was used and the result in **Figure 5** showed that curcumin supplement was safe for patients in experimental group compared with control group (OR=0.38, 95% CI [0.07 – 2.04], Z=1.13, P = 0.26; **Figure 6**).

## 4 Discussion

This systematic review and meta-analysis involved a total of ten studies and indicated that curcumin supplementation is one of the effective treatments for rheumatoid arthritis. The articles included in the analyses were generally different, but all passed through a complete statistical school. Although there was heterogeneity in the included articles, meta-analysis showed that factors such as patient age, rheumatoid arthritis history, serum abnormalities and threshold did not alter estimates of prognosis. The results of the current study showed that curcumin supplementation was effective in reducing clinical symptoms in the treatment of rheumatic diseases, and the use of curcumin supplementation in rheumatoid arthritis patients could reduce the possibility of unpleasant consequences.

Rheumatoid arthritis is this systemic inflammatory arthritis with a lifetime prevalence of up to 1% worldwide (29). Rheumatoid arthritis is an autoimmune disease and the pathophysiological mechanisms are complex, mainly affecting systemic joints and may present with varying degrees of extra-articular manifestations (30). It is characterized by a high incidence of cardiovascular disease, particularly in women and people with a history of smoking (31). Therefore, early diagnosis seems to be extremely important for the intervention and management of rheumatoid arthritis (32). It is expected to explain the pathogenesis of rheumatoid arthritis by focusing on novel genes and the close interaction between genetic factors and epigenetic mechanisms (33). It has been found that autoantibody isotypes and their molecular and physicochemical components may become potential therapeutic targets and strategies for the disease underlying immune processes (34). The response of some rheumatoid arthritis patients to antirheumatic drugs (DMARDs) remains limited in mitigating and preventing disease activity (35). Patients with rheumatoid arthritis tend to have extra-articular manifestations associated with more active and severe rheumatoid arthritis. The extra-articular features remain a major challenge in diagnosis and treatment for the disease, as they are associated with poor prognosis and required early recognition and timely management. The required effective alternative treatments are important to address this new challenge (36).

Curcumin, a pure natural compound derived from the roots of turmeric, is one of the most active components of polyphenolic curcuminoids (37). Zn (II) -curcumin compound has been found to exert anticancer effects on a variety of molecular mechanisms through Nrf2 regulation (38). Curcumin is effectively usedfor obesity therapy because it is a lipophilic molecule that can rapidly penetrate cell membranes, and may be associated with lipid metabolism, gut microbiota, and anti-inflammatory potential (39). Advanced encapsulation or loading of curcumin in the form of nanocurcumin has been shown to improve its therapeutic potential of multifaceted effects in the management of chronic diseases (40). It has been suggested that curcumin combined with photodynamic therapy could cause cell cycle arrest and inhibit apoptosis-mediating factor stimulation on disease cell viability, proliferation and migration (41). Curcumin is also known as “seasoning of life” by its well-known antibacterial, anti-inflammatory, antioxidant, antitumor, antifungal and pro-apoptotic effects (42).

Curcumin has been shown to improve symptoms and delay disease cycles in rheumatoid arthritis patients by inhibiting mitogen-activated protein kinase family, extracellular signal-regulated protein kinase, activator protein-1, and nuclear factor κB signal pathway in rheumatoid arthritis (43). Curcumin treated by rheumatoid arthritis patients with taking 250-1500 mg/day over 8-12 weeks can improve dysfunctional immune cells (including TH1, TH17, Treg and B cells) and reduce the clinical symptoms of the disease (44). It has been found that curcumin has therapeutic effects on collagen-induced arthritis (CIA) by inhibiting NF-κB signaling pathway and promoting macrophage apoptosis (45). Cur-TF combination with targeted delivery and enhanced efficacy of curcumin could improve clinical, histologic and radiographic scores, and reduce proinflammatory cytokines in rheumatoid arthritis patients (46). Curcumin has been found toaffect IL-10 secretion and immunoregulation to modulate the neurodegenerative diseases by promoting anti-inflammatory and immunosuppressive function (47). Curcumin supplementation enhances the anti-inflammatory capacity of the eicosanoid pathway in rheumatoid arthritis patients by modulating serum lipid levels to a normal state (48). It has been reported that curcumin improved the effect of fibroblast-like synoviocytes in rheumatoid arthritis by modulating the inc00052/miR-126-5p/PIAS2 axis and inhibiting Janus kinase 2 (JAK2)/signal transducer (49). Interestingly, curcumin suppressed inflammatory cell infiltration into synovium *via* rapamycin (mTOR) target pathway and suppressed the proinflammatory cytokine levels in rheumatoid arthritis rheumatoid arthritis (50). On the other hand, curcumin inhibits activation of PI3K/AKT signaling pathway and the expression of proinflammatory cytokines TNF-a, IL-6, and IL-17 (51). Therefore, curcumin can be used as a preventive measure in individuals at high risk of early or developing rheumatoid arthritis (52).

In this systematic review and meta-analysis, we found that curcumin could improve ESR, CRP, DAS, RF, VAS, TJC, and SJC in patients with rheumatoid arthritis. Our findings suggest that rheumatoid arthritis patients in the experimental group supplemented with curcumin benefit more than patients in the control group. For rheumatoid arthritis patients, functional improvement of total proteins by curcumin is uncertain. The current study suggest that curcumin supplementation may be an effective method to enhance anti-rheumatic immunity. In our study, no serious adverse events were found in ten studies. The mild side effects such as constipation, diarrhea, rash, nausea, and vomiting may occur during intervention with curcumin. No statistically significant difference was found in the curcumin group and the control group, suggesting that curcumin supplementation is safe for intervention in rheumatoid arthritis.

In our study, high heterogeneity was found and then the random-effects model was used. The causes of the heterogeneity were complex and mainly divided into the following types: clinical heterogeneity, ethnic heterogeneity and methodological heterogeneity. In our study, most of the results in the experimental group were statistically significant. It was also clear that curcumin has great potential in the treatment of rheumatoid arthritis, overcoming the dosing barriers through nano/submicron carrier-based delivery techniques and maximizing the efficacy of anti-rheumatic drugs.

This research has several restrictions: (1) Although ten RCTs were included in this study, the sample size was relatively small; (2) Most of the included RCTs did not report long-term effects of drugs and the prognosis of patients; (3) Some included original studies used unified interventions and did not distinguish patients by syndrome. (4) Although the study used total protein as a secondary outcome measure, the literature is sparse.

## 5 Conclusion

Modern medical research has found that the occurrence of many diseases in the human body is related to free radical formation and inflammatory response. The multiple double bonds in curcumin endow it with trapping free radical electrons, antioxidant activity and anti-inflammatory effects. Curcumin is beneficial for rheumatoid arthritis treatment. Inflammation levels and clinical symptoms in patients with rheumatoid arthritis can be improved by curcumin supplementation. Large sample randomized controlled trials on the effects of curcumin on patients with rheumatoid arthritis are needed in the future.

## Data availability statement

The original contributions presented in the study are included in the article/**Supplementary Material**. Further inquiries can be directed to the corresponding authors.

## Author contributions

HK and LH contributed equally to this study. All authors contributed to the work and approved the final manuscript.

## References

[1] Akiyama M Kaneko Y . Pathogenesis, clinical features, and treatment strategy for rheumatoid arthritis-associated interstitial lung disease. Autoimmun Rev (2022) 21(5):103056. doi: 10.1016/j.autrev.2022.103056 35121155

[2] Liu D Zhang F Cao H Wang X . Can sexual dimorphism in rheumatoid arthritis be attributed to the different abundance of Gardnerella? Ann Rheum Dis (2022) 81(3):e36. doi: 10.1136/annrheumdis-2020-217214 32156707

[3] Mizuuchi T Sawada T Nishiyama S Tahara K Hayashi H Mori H . Distal interphalangeal joint involvement may be associated with disease activity and affected joint distribution in rheumatoid arthritis. J Clin Med (2022) 11(5):76-7. doi: 10.3390/jcm11051405 PMC8911492 35268496

[4] Gadeval A Chaudhari S Bollampally SP Polaka S Kalyane D Sengupta P . Integrated nanomaterials for non-invasive photothermal therapy of rheumatoid arthritis. Drug Discovery Today (2021) 26(10):2315–28. doi: 10.1016/j.drudis.2021.04.026 33962037

[5] Itaya T Torii M Hashimoto M Tanigawa K Urai Y Kinoshita A . Prevalence of anxiety and depression in patients with rheumatoid arthritis before and during the COVID-19 pandemic. Rheumatol (Oxford) (2021) 60(4):2023–4. doi: 10.1093/rheumatology/keab065 PMC7928586 33502497

[6] Nygaard G Firestein GS . Restoring synovial homeostasis in rheumatoid arthritis by targeting fibroblast-like synoviocytes. Nat Rev Rheumatol (2020) 16(6):316–33. doi: 10.1038/s41584-020-0413-5 PMC7987137 32393826

[7] Soussi BG Cordtz RL Kristensen S Bork CS Christensen JH Schmidt EB . Incidence and prevalence of rheumatoid arthritis in Denmark from 1998 to 2018: a nationwide register-based study. Scand J Rheumatol (2021) 51(6):481–9. doi: 10.1080/03009742.2021.1957557 34913402

[8] Burmester GR Pope JE Liang C Wang S Owusu Boadi E . Novel treatment strategies in rheumatoid arthritis. Lancet (2017) 389(10086):2338–48. doi: 10.1016/S0140-6736(17)31491-5 28612748

[9] Smolen JS Aletaha D Mcinnes IB . Rheumatoid arthritis. Lancet (2016) 388(10055):2023–38. doi: 10.1016/S0140-6736(16)30173-8 27156434

[10] Rech J Schett G . Towards preventive treatment of rheumatoid arthritis. Lancet (2022) 400(10348):253–5. doi: 10.1016/S0140-6736(22)01327-7 35871801

[11] Lin YJ Anzaghe M Schülke S . Update on the pathomechanism, diagnosis, and treatment options for rheumatoid arthritis. Cells (2020) 9(4):97-100. doi: 10.3390/cells9040880 PMC7226834 32260219

[12] Bermas BL . Paternal safety of anti-rheumatic medications. Best Pract Res Clin Obstet Gynaecol (2020) 64:77–84. doi: 10.1016/j.bpobgyn.2019.09.004 31727565

[13] Wang Y Chen S Du K Liang C Wang S Owusu Boadi E . Traditional herbal medicine: therapeutic potential in rheumatoid arthritis. J Ethnopharmacol (2021) 279:114368. doi: 10.1016/j.jep.2021.114368 34197960

[14] Hewlings SJ Kalman DS . Curcumin: a review of its effects on human health. Foods (2017) 6(10):108-11. doi: 10.3390/foods6100092 29065496 PMC5664031

[15] Anand P Kunnumakkara AB Newman RA Aggarwal BB . Bioavailability of curcumin: problems and promises. Mol Pharm (2007) 4(6):807–18. doi: 10.1021/mp700113r 17999464

[16] Zeng L Yang T Yang K Yu G Li J Xiang W . Curcumin and curcuma longa extract in the treatment of 10 types of autoimmune diseases: a systematic review and meta-analysis of 31 randomized controlled trials. Front Immunol (2022) 13:896476. doi: 10.3389/fimmu.2022.896476 35979355 PMC9376628

[17] Ju DT Tsai BC Sitorus MA Kuo WW Kuo CH Chen TS . Curcumin-pretreated adipose-derived stem cells enhance the neuroprotective ability to repair rheumatoid arthritis-induced damage in the rat brain. Am J Chin Med (2022) 50(5):1299–314. doi: 10.1142/S0192415X22500549 35726142

[18] Cook DA Reed DA . Appraising the quality of medical education research methods: the medical education research study quality instrument and the Newcastle-Ottawa scale-education. Acad Med (2015) 90(8):1067–76. doi: 10.1097/ACM.0000000000000786 26107881

[19] Amalraj A Varma K Jacob J Divya C Kunnumakkara AB Stohs SJ . A novel highly bioavailable curcumin formulation improves symptoms and diagnostic indicators in rheumatoid arthritis patients: a randomized, double-blind, placebo-controlled, two-dose, three-arm, and parallel-group study. J medicinal Food (2017) 20(10):1022–30. doi: 10.1089/jmf.2017.3930 28850308

[20] Chandran B Goel A . A randomized, pilot study to assess the efficacy and safety of curcumin in patients with active rheumatoid arthritis. Phytotherapy research: PTR (2012) 26(11):1719–25. doi: 10.1002/ptr.4639 22407780

[21] Pourhabibi-Zarandi F Rafraf M Zayeni H Asghari-Jafarabadi M Ebrahimi AA . Effects of curcumin supplementation on metabolic parameters, inflammatory factors and obesity values in women with rheumatoid arthritis: a randomized, double-blind, placebo-controlled clinical trial. Phytotherapy research: PTR (2022) 36(4):1797–806. doi: 10.1002/ptr.7422 35178811

[22] Javadi M Khadem Haghighian H Goodarzy S Goodarzy S Abbasi M Nassiri-Asl M . Effect of curcumin nanomicelle on the clinical symptoms of patients with rheumatoid arthritis: a randomized, double-blind, controlled trial. Int J rheumatic Dis (2019) 22(10):1857–62. doi: 10.1111/1756-185X.13688 31482684

[23] Deodhar SD Sethi R Srimal RC . Preliminary study on antirheumatic activity of curcumin (diferuloyl methane). Indian J Med Res (1980) 71:632–4. 7390600

[24] Hemmati AA Rajaee E Houshmand G Fakhroddin MA Dargahi-Malamir M Hesam S . Study the effects of anti-inflammatory curcumex capsules containing three plants (ginger, curcumin and black pepper) in patients with active rheumatoid arthritis. IIOAB J (2016) 7:389–92. Available at: https://www.iioab.org/articles/IIOABJ_7.S5_389-392.pdf.

[25] Khan MK Khan IA Liaquat A . Therapeutic potential of curcumin with and without strengthening exercises in improving rheumatoid arthritis. J Coll Physicians Surg Pak (2022) 32(12):1640–3. doi: 10.29271/jcpsp.2022.12.1640 36474395

[26] Anusha D Chaly PE Junaid M Nijesh Shivashankar JE Sivasamy S . Efficacy of a mouthwash containing essential oils and curcumin as an adjunct to nonsurgical periodontal therapy among rheumatoid arthritis patients with chronic periodontitis: a randomized controlled trial. Indian J Dent Res (2019) 30(4):506–11. doi: 10.4103/ijdr.IJDR_662_17 31745043

[27] Lin JYH Wang T . Curcumin combined with methotrexate in the treatment of rheumatoid arthritis bone destruction. Tianjin Tradit Chin Med (2019) 36(03):238–41. doi: 10.11656/j.issn.1672-1519.2019.03.08

[28] Jacob J Amalraj A Raj KKJ Divya C Kunnumakkara AB Gopi S . A novel bioavailable hydrogenated curcuminoids formulation (CuroWhite™) improves symptoms and diagnostic indicators in rheumatoid arthritis patients - a randomized, double blind and placebo controlled study. J Tradit Complement Med (2019) 9(4):346–52. doi: 10.1016/j.jtcme.2018.06.001 PMC6702143 31453131

[29] Wasserman A . Rheumatoid arthritis: common questions about diagnosis and management. Am Fam Physician (2018) 97(7):455–62. 29671563

[30] Radu AF Bungau SG . Management of rheumatoid arthritis: an overview. Cells (2021) 10(11):293-5. doi: 10.3390/cells10112857 34831081 PMC8616326

[31] England BR Thiele GM Anderson DR Mikuls TR . Increased cardiovascular risk in rheumatoid arthritis: mechanisms and implications. Bmj (2018) 361:k1036. doi: 10.1136/bmj.k1036 29685876 PMC6889899

[32] Cush JJ . Rheumatoid arthritis: early diagnosis and treatment. Med Clin North Am (2021) 105(2):355–65. doi: 10.1016/j.mcna.2020.10.006 33589108

[33] Croia C Bursi R Sutera D Petrelli F Alunno A Puxeddu I . One year in review 2019: pathogenesis of rheumatoid arthritis. Clin Exp Rheumatol (2019) 37(3):347–57. 31111823

[34] Van Delft MAM Huizinga TWJ . An overview of autoantibodies in rheumatoid arthritis. J Autoimmun (2020) 110:102392. doi: 10.1016/j.jaut.2019.102392 31911013

[35] Huang J Fu X Chen X Li Z Huang Y Liang C . Promising therapeutic targets for treatment of rheumatoid arthritis. Front Immunol (2021) 12:686155. doi: 10.3389/fimmu.2021.686155 34305919 PMC8299711

[36] Conforti A Di Cola I Pavlych V Ruscitti P Berardicurti O Ursini F . Beyond the joints, the extra-articular manifestations in rheumatoid arthritis. Autoimmun Rev (2021) 20(2):102735. doi: 10.1016/j.autrev.2020.102735 33346115

[37] Jabczyk M Nowak J Hudzik B Zubelewicz-Szkodzińska B . Curcumin in metabolic health and disease. Nutrients (2021) 13(12):311-2. doi: 10.3390/nu13124440 34959992 PMC8706619

[38] Shahcheraghi SH Salemi F Peirovi N Ayatollahi J Alam W Khan H . Nrf2 regulation by curcumin: molecular aspects for therapeutic prospects. Molecules (2021) 27(1):312-4 doi: 10.3390/molecules27010167 PMC8746993 35011412

[39] Kasprzak-Drozd K Oniszczuk T Gancarz M Kondracka A Rusinek R Oniszczuk A . Curcumin and weight loss: does it work? Int J Mol Sci (2022) 23(2):314-7. doi: 10.3390/ijms23020639 PMC8775659 35054828

[40] Tagde P Tagde P Islam F Tagde S Shah M Hussain ZD . The multifaceted role of curcumin in advanced nanocurcumin form in the treatment and management of chronic disorders. Molecules (2021) 26(23):317-9. doi: 10.3390/molecules26237109 34885693 PMC8659038

[41] Xie L Ji X Zhang Q Wei Y . Curcumin combined with photodynamic therapy, promising therapies for the treatment of cancer[J]. BioMed Pharmacother (2022) 146:112567. doi: 10.1016/j.biopha.2021.112567 34953392

[42] Benameur T Giacomucci G Panaro MA Ruggiero M Trotta T Monda V . New promising therapeutic avenues of curcumin in brain diseases. Molecules (2021) 27(1):322-4. doi: 10.3390/molecules27010236 PMC8746812 35011468

[43] Pourhabibi-Zarandi F Shojaei-Zarghani S Rafraf M . Curcumin and rheumatoid arthritis: a systematic review of literature. Int J Clin Pract (2021) 75(10):e14280. doi: 10.1111/ijcp.14280 33914984

[44] Mohammadian Haftcheshmeh S Khosrojerdi A Aliabadi A Lotfi S Mohammadi A Momtazi-Borojeni AA . Immunomodulatory effects of curcumin in rheumatoid arthritis: evidence from molecular mechanisms to clinical outcomes. Rev Physiol Biochem Pharmacol (2021) 179:1–29. doi: 10.1007/112_2020_54 33404796

[45] Wang Q Ye C Sun S Li R Shi X Wang S . Curcumin attenuates collagen-induced rat arthritis *via* anti-inflammatory and apoptotic effects. Int Immunopharmacol (2019) 72:292–300. doi: 10.1016/j.intimp.2019.04.027 31005039

[46] Sana E Zeeshan M Ain QU Khan AU Hussain I Khan S . Topical delivery of curcumin-loaded transfersomes gel ameliorated rheumatoid arthritis by inhibiting NF-κβ pathway. Nanomedicine (Lond) (2021) 16(10):819–37. doi: 10.2217/nnm-2020-0316 33900118

[47] Mollazadeh H Cicero AFG Blesso CN Pirro M Majeed M Sahebkar A . Immune modulation by curcumin: the role of interleukin-10. Crit Rev Food Sci Nutr (2019) 59(1):89–101. doi: 10.1080/10408398.2017.1358139 28799796

[48] Yang M Akbar U Mohan C . Curcumin in autoimmune and rheumatic diseases. Nutrients (2019) 11(5):338-40. doi: 10.3390/nu11051004 31052496 PMC6566522

[49] Xiao J Cai X Zhou W Wang R Ye Z . Curcumin relieved the rheumatoid arthritis progression *via* modulating the linc00052/miR-126-5p/PIAS2 axis. Bioengineered (2022) 13(4):10973–83. doi: 10.1080/21655979.2022.2066760 PMC9208441 35473503

[50] Dai Q Zhou D Xu L Song X . Curcumin alleviates rheumatoid arthritis-induced inflammation and synovial hyperplasia by targeting mTOR pathway in rats. Drug Des Devel Ther (2018) 12:4095–105. doi: 10.2147/DDDT.S175763 PMC6284537 30584274

[51] Xu Z Shang W Zhao Z Zhang B Liu C Cai H . Curcumin alleviates rheumatoid arthritis progression through the phosphatidylinositol 3-kinase/protein kinase b pathway: an *in vitro* and *in vivo* study. Bioengineered (2022) 13(5):12899–911. doi: 10.1080/21655979.2022.2078942 PMC9276000 35609329

[52] Asteriou E Gkoutzourelas A Mavropoulos A Katsiari C Sakkas LI Bogdanos DP . Curcumin for the management of periodontitis and early ACPA-positive rheumatoid arthritis: killing two birds with one stone. Nutrients (2018) 10(7):347-9. doi: 10.3390/nu10070908 30012973 PMC6073415

