# Supplementary figures and images for: Effect of curcumin on rheumatoid arthritis: a systematic review and meta-analysis
Source: Front Immunol. 2023 May 31;14:1121655. doi: 10.3389/fimmu.2023.1121655 (PMC10264675; doi:10.3389/fimmu.2023.1121655)

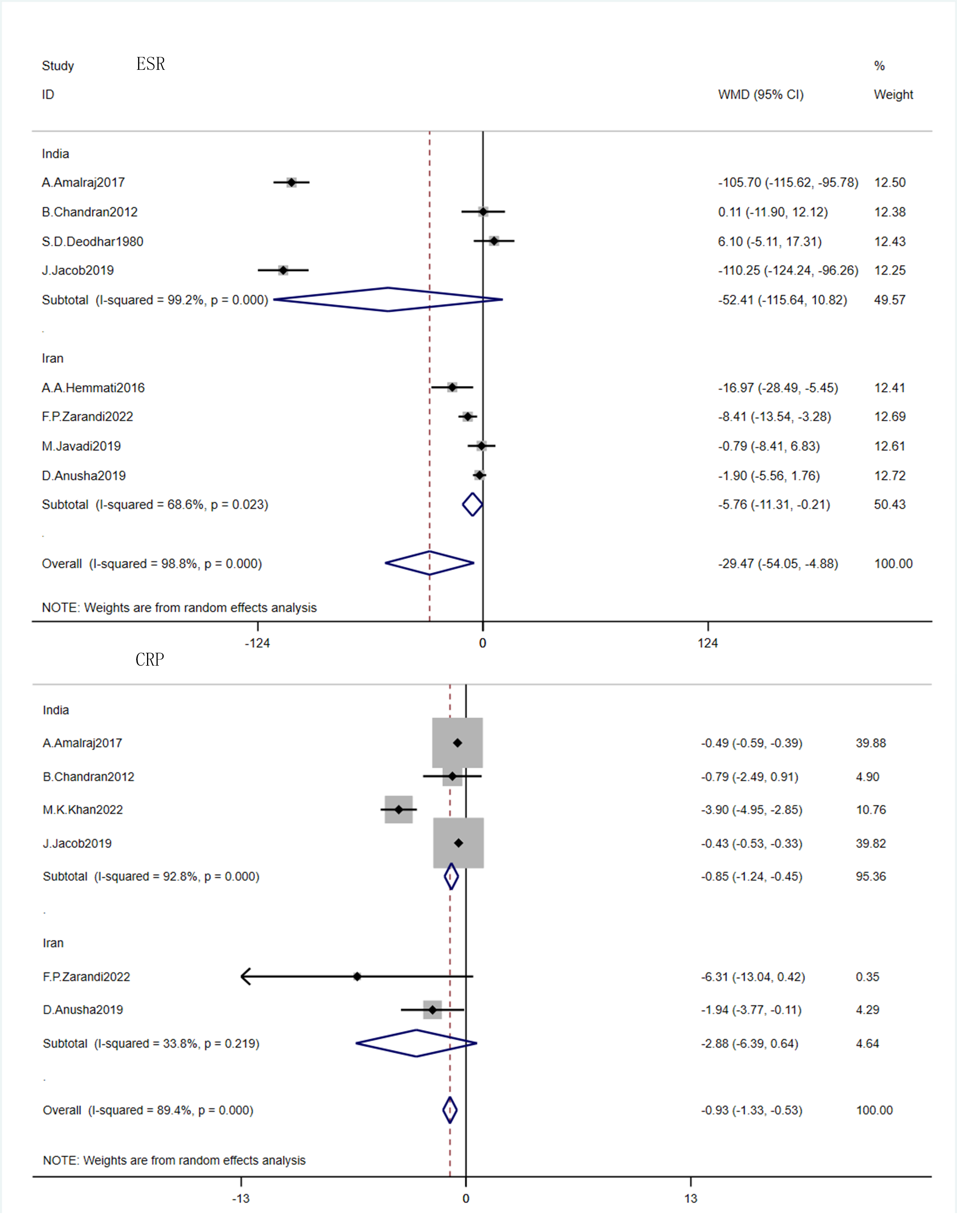

Supplement: Supplementary file 1 [file Image_1.tif]

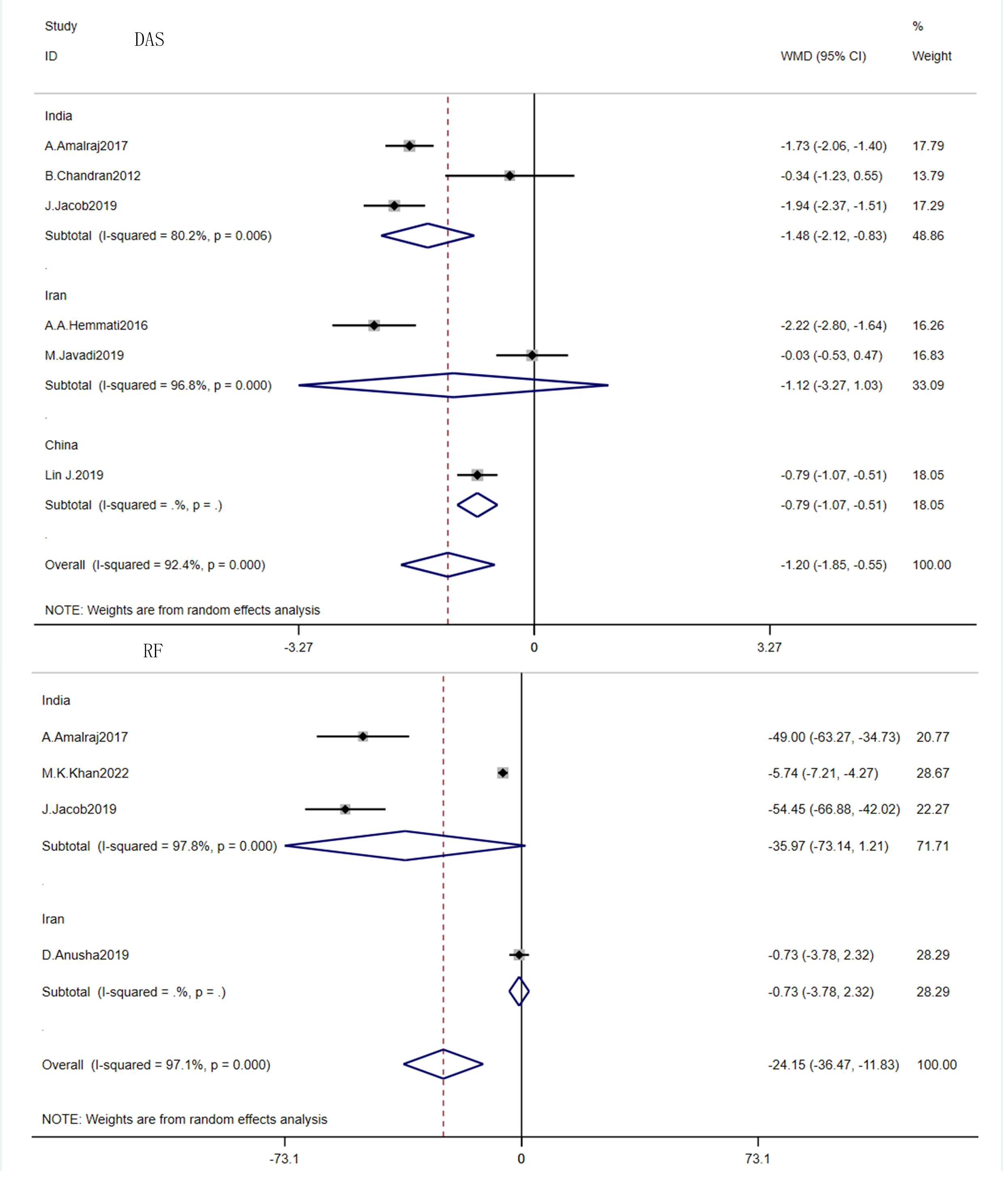

Supplement: Supplementary file 2 [file Image_2.tif]

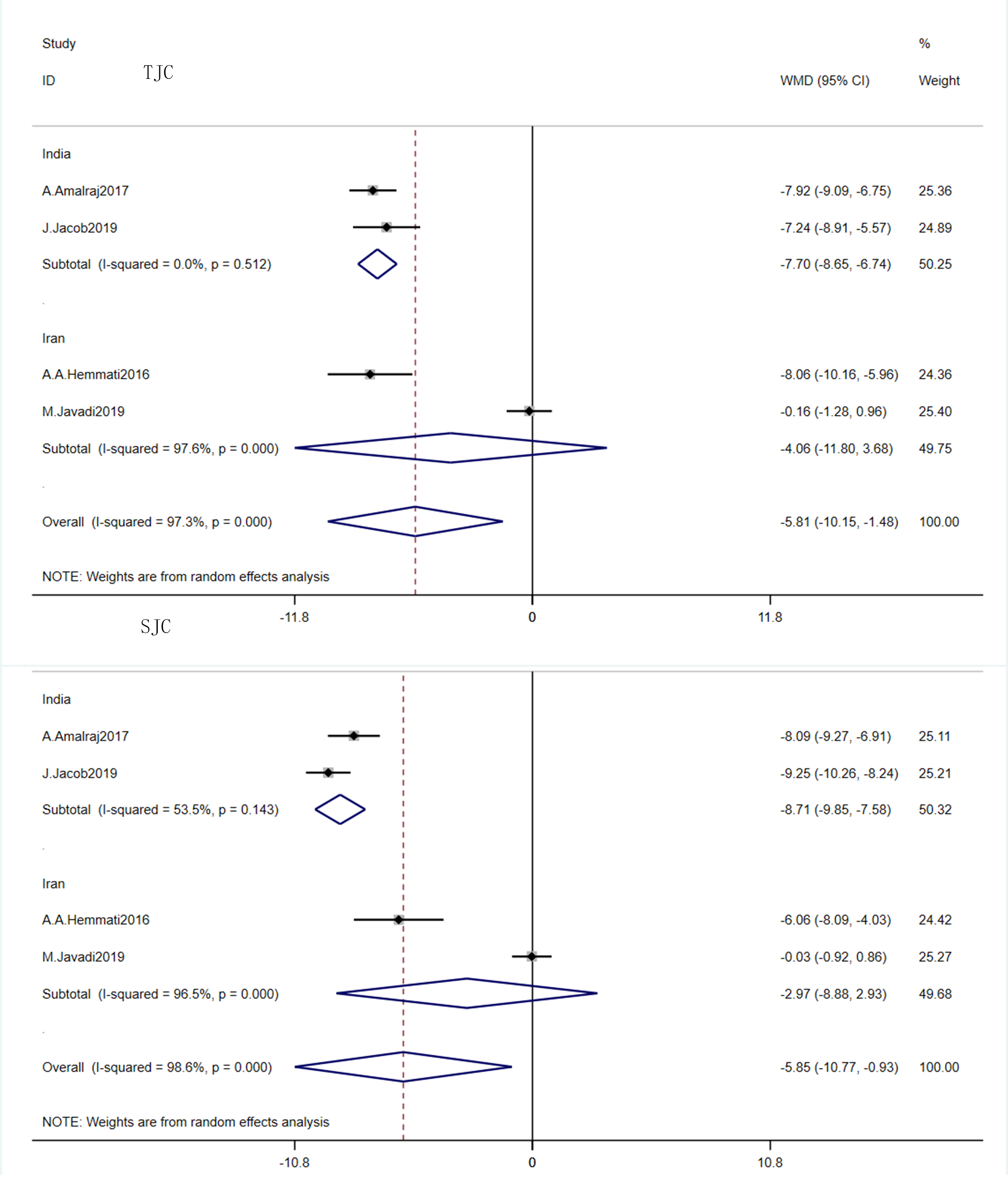

Supplement: Supplementary file 3 [file Image_3.tif]

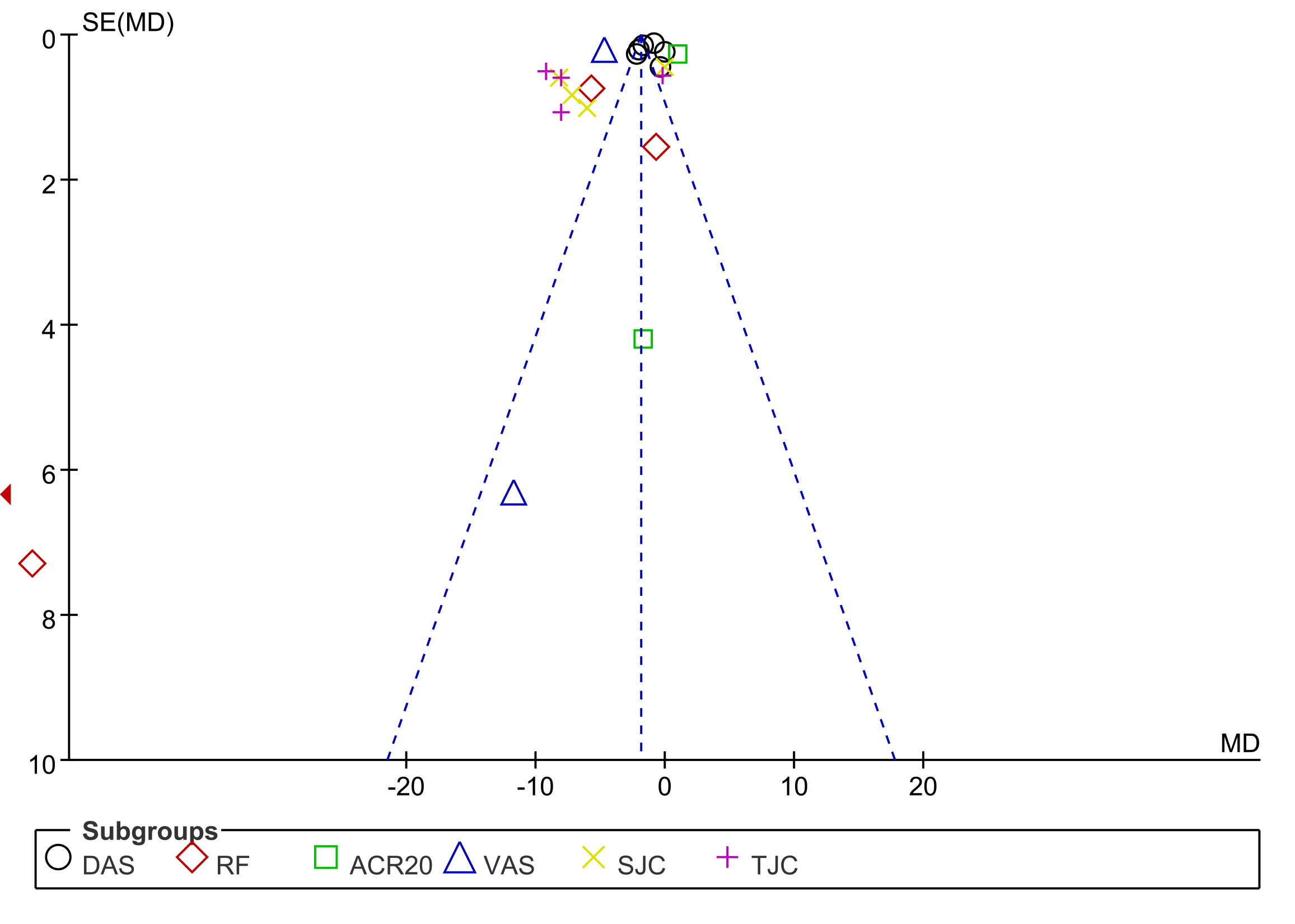

Supplement: Supplementary file 4 [file Image_4.tif]
